# HIV-TAT mediated protein transduction of Cu/Zn-superoxide dismutase-1 (SOD_1_) protects skin cells from ionizing radiation

**DOI:** 10.1186/1748-717X-8-253

**Published:** 2013-10-31

**Authors:** Qing Gu, Tienan Feng, Han Cao, Yiting Tang, Xin Ge, Judong Luo, Jiao Xue, Jinyong Wu, Hongying Yang, Shuyu Zhang, Jianping Cao

**Affiliations:** 1 School of Radiation Medicine and Protection and Jiangsu Provincial Key Laboratory of Radiation Medicine and Protection, Soochow University, Suzhou 215123, China; 2 Department of Disaster and Emergency Medicine, Eastern Hospital, Tongji University School of Medicine, Shanghai 200120, China; 3 BD Rapid Diagnostics (Suzhou) Co., Ltd., Suzhou 215000, China; 4 Department of Radiotherapy, Changzhou Tumor Hospital, Soochow University, Changzhou 213001, China; 5 Institute of Plasma Physics, Chinese Academy of Sciences, Hefei 230031, China

**Keywords:** Cu/Zn-superoxide dismutase (SOD1), Protein transduction domain (PTD), HIV-TAT domain, Radiation-induced skin injury

## Abstract

**Background:**

Radiation-induced skin injury remains a serious concern during radiotherapy. Cu/Zn-superoxide dismutase (Cu/Zn-SOD, SOD_1_) is a conserved enzyme for scavenging superoxide radical in cells. Because of the integrity of cell membranes, exogenous molecule is not able to be incorporated into cells, which limited the application of natural SOD_1_. The aim of this study was to evaluate the protective role of HIV-TAT protein transduction domain mediated protein transduction of SOD_1_ (TAT-SOD_1_) against ionizing radiation.

**Methods:**

The recombinant TAT-SOD_1_ and SOD_1_ were obtained by prokaryotic–based protein expression system. The transduction effect and biological activity of TAT-SOD_1_ was measured by immunofluorescence and antioxidant capability assays in human keratinocyte HaCaT cells. Mito-Tracker staining, reactive oxygen species (ROS) generation assay, cell apoptosis analysis and malondialdehyde (MDA) assay were used to access the protective effect of TAT- SOD_1_.

**Results:**

Uptake of TAT-SOD_1_ by HaCaT cells retained its biological activity. Compared with natural SOD_1_, the application of TAT-SOD_1_ significantly enhanced the viability and decreased the apoptosis induced by X-ray irradiation. Moreover, TAT-SOD_1_ reduced ROS and preserved mitochondrial integrity after radiation exposure in HaCaT cells. Radiation-induced γH2AX foci, which are representative of DNA double strand breaks, were decreased by pretreatment with TAT-SOD_1_. Furthermore, subcutaneous application of TAT-SOD_1_ resulted in a significant decrease in 45 Gy electron beam-induced ROS and MDA concentration in the skins of rats.

**Conclusions:**

This study provides evidences for the protective role of TAT-SOD_1_ in alleviating radiation-induced damage in HaCaT cells and rat skins, which suggests a new therapeutic strategy for radiation-induced skin injury.

## Introduction

Radiation therapy is widely used for the treatment of various types of cancers [1]. However, following the destruction of tumors, surrounding normal tissues may also be injured, including brain, lung, intestine and skin etc. Because skin is usually the first site of external radiation particles entry in radiation treatment, variable degrees of skin reactions can occur. Serious radiation-induced skin injuries can cause severe pain, deformation, secondary infection, ulceration, and even necrosis when intolerable doses are administered [2]. Ionizing radiation (IR) is known to induce reactive oxidative species (ROS) production due to radiolysis of water and direct ionization of target molecules, which could result in oxidative damage and cytotoxicity to critical cellular biomolecules, including nucleic acids, proteins, and lipids [3].

Cells have involved a system to counteract radiation-induced ROS. Superoxide dismutases (SODs) are enzymes that catalyze the degradation of superoxide into oxygen and hydrogen peroxide. The generated hydrogen peroxide is subsequently converted to water and oxygen by glutathione peroxidase (GPx) and/or catalase (CAT). Three types of SOD isozymes have been identified in human cells and Cu/Zn-superoxide dismutase (SOD_1_) contributes to approximately 70–80% of cellular SOD activity. SOD_1_ is a 20-kDa homodimer antioxidant enzyme, which is widely distributed in the cytosol, nucleus, peroxisome, and intermembrane space of mitochondria in mammalian cells [4,5]. Insufficient supply of intracellular antioxidant enzymes, especially SOD_1_ can cause overproduction of ROS, leading to apoptosis and necrosis, eventually cell death [6]. Although this antioxidant enzyme has been recognized available to protect cells against oxidative stress induced by ionizing radiation, the application of SOD_1_ was limited, because their hydrophilic properties and large molecular size that prevents it from entering target cells [7].

Protein transduction domains (PTDs), also known as cell penetrating peptides, have been shown to deliver proteins across cell membranes and applied for numerous diseases as protein therapy [8,9]. The human immunodeficiency virus type 1 (HIV) transactivator of transcription (TAT) domain is a regulatory element that drastically enhances the efficiency of viral transcription. It has been identified as the first member of PTD superfamily, which can deliver heterologous proteins across most biomembranes without losing their bioactivity [10]. In 1988, two groups independently characterized the HIV TAT as a PTD, which allows TAT to enter cells by crossing the cell membrane. The essential amino acid sequence of the TAT PTD has been characterized as YGRKKRRQRRR [11,12]. In 1994, Fawell *et al*. confirmed that the TAT protein can carry exogenous molecules into cells [13]. When fused with PTDs by gene-recombinant expression, exogenous proteins can be delivered across the cell membrane via endocytosis [14-16]. The TAT PTD has emerged as a promising tool for non-invasive cellular delivery of cargos and has been successfully applied to *in vitro* and *in vivo* delivery of a variety of therapeutic agents for the treatment of multiple diseases [17,18]. Furthermore, TAT PTD linked-SOD_1_ was found to be delivered not only into the cytoplasm but also into mitochondria where superoxide is generated, suggesting a dramatic potential of TAT-SOD_1_ as an ideal intracellular antioxidant solution [19]. However, it is not clear whether TAT-SOD_1_ has protective therapeutic role against radiation-induced skin injury. In this study, we prepared TAT-SOD_1_ recombinant protein and examined its protective effects on skin damage induced by ionizing radiation.

## Materials and methods

### Reagents

Mito-Tracker Mitochondrion-selective Probes (Mito-Tracker Red CM-H2XRos) and 2′,7′-Dichlorofluorescin diacetate (DCF-DA) were purchased from Invitrogen (Carlsbad, CA, USA). Primary antibodies against SOD_1_ and β-actin (Santa Cruz Biotechnology, Santa Cruz, CA, USA), and γH2AX (Epitomics, Burlingame, CA, USA) were obtained commercially. HRP-conjugated anti-rabbit or anti-mouse secondary antibodies were supplied by Santa Cruz Biotechnology (Santa Cruz, CA, USA).

### Cell culture and irradiation

Human epidermal keratinocyte cell line HaCaT was maintained in Dulbecco’s modified Eagle’s medium (DMEM) supplemented with 10% (v/v) heat-inactivated fetal bovine serum and 1% (v/v) penicillin–streptomycin at 37°C in a humidified atmosphere containing 5% CO_2_. Briefly, exponentially growing cells were exposed to different dosages (5 Gy or 20 Gy) of ionizing radiation using X-ray linear accelerator (Rad Source, Suwanee, GA, USA) at a fixed dose rate of 1.15Gy/min. Sham irradiated cells were defined as 0 Gy group. 20 Gy X-ray was chosen because it induces significant cell death [20]. 5 Gy X-ray was chose because it causes appropriate DNA damage [21]. HaCaT cells were pretreated with PBS, SOD_1_ or TAT-SOD_1_ 5 h before X-ray irradiation or sham irradiation. Cells treated with PBS were considered as negative controls.

### Construction of the TAT-SOD_1_ expression vector

**Figure 1:**
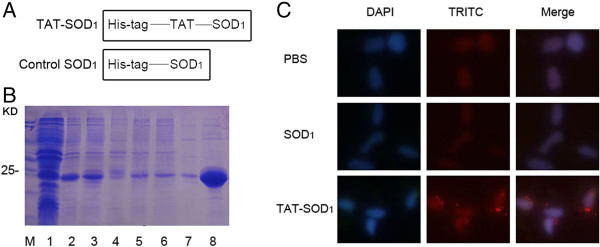
**Expression and purification of TAT-SOD**_**1**_**protein. (A)** Schematic representation of the TAT-SOD_1_ protein. **(B)** Expressed and purified fusion proteins were analyzed by 12% SDS-PAGE followed by Coomassie Blue staining. Lanes in B are as follows: lane 1, non-induced TAT-SOD_1_; lane 2, induced TAT-SOD_1_; lane 3, supernatant after sonication; lane 4, precipitation after sonication; lane 5, Effluent after loading;lane6, washing solution; lane 7, eluent buffer 1; lane 8, eluent buffer 2. **(C)** Purified fusion proteins were subjected to immunofluorescence analysis with an anti-rabbit SOD_1_ antibody.

The coding sequence of the HIV-TAT domain (YGRKKRRQRRR) was synthesized and subcloned into the *BamHI* and *EcoRI* sites of pET-28a (Novagen, Madison, WI, USA) to generate pET-28a-TAT. Human SOD_1_ cDNA was amplified by PCR using appropriate primers and then subcloned into the *EcoR*I and *Xho*I sites of pET-28a-TAT to generate pET-28a-SOD_1_ (Figure 1A). The control vector, which expressed SOD_1_, was constructed by inserting SOD_1_ coding sequence into pET-28a without TAT domain.

### Purification of target fusion protein

Host *E. coli* BL21 (DE3) were transformed with the plasmid encoding pET-28a-SOD_1_, and then the transformants were selected on a LB plate containing kanamycin. BL21 (DE3) cells containing the expression plasmid were grown at 37°C to an optical density OD_600_ of 0.8. Isopropyl-beta-D-thiogalactoside (IPTG) was added to a final concentration of 1.0 mM, and the cells were then incubated for an additional 8 h at 25°C. Cells were sonicated, and the supernatants were recovered and applied to a column of Ni-nitrilotriacetic acid agarose (Qiagen, Valencia, CA, USA). Then, the mixture was maintained at 4°C with shaking at 50 rpm. After the TAT-SOD_1_ had been absorbed by the column, the column was settled onto a plastic filter by gravity. After the Ni^2+^ column had settled to the bottom, the resin was washed twice with 4 ml washing buffer (10 mM imidazole, 0.5 M NaCl, and 50 mM NaH_2_PO_4_, pH 8.0). Then, the fusion protein was eluted with elution buffer (150 mM imidazole, 0.3 M NaCl, and 50 mM NaH_2_PO_4_, pH 8.0). The fractions containing fusion proteins were combined.

### Immunofluorescence assay

HaCaT cells were plated on glass coverslips in six-well plates for 12 h, and then incubated with fresh medium containing PBS, SOD_1_ or TAT-SOD_1_ for 5 h at 37°C in a 5% CO_2_ atmosphere. The cells were washed with PBS, fixed using freshly prepared 4% paraformaldehyde and permeabilized with 0.5% Triton X-100 (Sigma, St. Louis, MO, USA) after treatment. The SOD_1_ antibody (Santa Cruz Biotechnology, Santa Cruz, CA, USA) diluted 1:500 in 1% bovine serum albumin (BSA, Solarbio, Beijing, China) in PBS and incubated for 12 h at 4°C, followed by incubation with the secondary antibody, fluorescein TRITC-conjugated goat anti-rabbit IgG (Southern Biotech, Birmingham, AL, USA) for 45 min at 37°C. The cells were counter stained using DAPI to visualize the cell nucleus and were observed using a fluorescence microscope.

### Total antioxidant capability assay, SOD enzyme assay and superoxide scavenging activity assay

HaCaT cells were grown to confluence on 6-well plates, after treatment with PBS, SOD_1_, TAT-SOD_1_ for 5 h, cells were washed with PBS, exposed to trypsin without EDTA for 10 min and washed again with PBS. The cells were harvested for preparation of cell extracts to determine total anti-oxidation competence (T-AOC) assay, SOD enzyme assay and superoxide scavenging activity assay (all from Nanjing Jiancheng Bioengineering Institute, Nanjing, China). Protein concentration of cell lysate was detected by BCA assay kit (Sigma, St. Louis, MO, USA).

### Cell viability assay

After centrifuging and suspension, the HaCaT cells were seeded in 96-well plates at 4000 cells/well. The cells were treated with PBS, SOD_1_ or TAT-SOD_1_ 5 h before 20 Gy irradiation. 48 h later, CCK-8 solution (Dojindo Molecular Technologies Inc. Kumamoto, Japan) was added and the plates were placed in a CO_2_ incubator for additional 2 h according to the manufacturer’s instructions. The optical density (OD) was determined at 450 nm. Each group was set up in triplicate.

### Cell apoptosis assay

We used two methods to detect the cell apoptosis, DNA fragmentation and Annexin V- propidium iodide (PI) double staining analysis. Effects of transduced TAT-SOD_1_ on DNA fragmentation in HaCaT cells exposed to 20 Gy ionizing radiation. PBS, SOD_1_ or TAT-SOD_1_ was added to cultured cells 5 h before irradiation, respectively. And then cells were further incubated for 48 h, followed by DNA extraction. Genomic DNA extraction kit was purchased from TianGen (Beijing, China). Isolated DNA, mainly derived from the apoptotic bodies occurred in cells, was subjected to 1.2% agarose electrophoresis at 35 V for 4 h. HaCaT cells were seeded at 1 × 10^5^ per well in 6-well plates. For Annexin V- and PI double staining analysis, cells were treated with SOD_1_ or TAT-SOD_1_ for 5 h before 20 Gy irradiation. After 48 h, cells were stained with fluorescein FITC-conjugated Annexin V and PI (KeyGen, Nanjing, China). The analysis was performed using flow cytometry (Beckman-Coulter, Brea, CA, USA).

### Western blot

Lysates from HaCaT cells were prepared by incubating cells in lysis buffer (125 mM Tris–HCl, pH 6.8, 2% SDS, 10% v/v, glycerol.) at 4°C for 30 min. Protein concentration was determined by BCA protein assay (Bio-Rad Laboratories, Hercules, CA, USA). 40 μg of cell lysates were subjected to 12% sodium dodecyl sulfate-polyacrylamide gel electrophoresis (SDS-PAGE). Following SDS-PAGE, the proteins were transferred onto a PVDF membrane (Millipore, Temecula, CA, USA), which was blocked with 10% dry milk in TBST. The membrane was then probed with the indicated antibodies at 4°C for 12 h, followed by HRP-conjugated anti-rabbit or mouse IgG secondary antibody (Santa Cruz Biotechnology, Santa Cruz, CA, USA). The immunoreactive bands were detected by chemiluminescence using an ECL system (Amersham, Chicago, IL, USA).

### Mitochondria integrity assay

Mito-Tracker was used to determine the mitochondria integrity of HaCaT cells. HaCaT cells were planted in 35 mm glass dishes. 24 h later, PBS, SOD_1_ or TAT-SOD_1_ was added in the dishes 5 h before 20 Gy X-ray irradiation. After treatment, cells were incubated for 30 min in the dark with Mito-Tracker red fluorescent stain which was dissolved in serum-free medium at 37°C. Hoechst stains were used to mark the nucleus.

### Reactive oxygen species generation assay

ROS levels of HaCaT were determined using the ROS sensitive dye 2′,7′-dichlorofluorescein diacetate (DCF-DA), which is converted by ROS into the highly fluorescent 2′,7′-dichlorofluorescein (DCF). After treatment, HaCaT cells were washed with phosphate buffer (pH 7.4) and incubated with DCF-DA (10 μM) for 30 min. The level of DCF fluorescence, reflecting the concentration of ROS, was measured by a fluorescence microscope. Fluorescence intensity of each group was analyzed by ImageJ image analysis software (NIH, Bethesda, MD, USA).

For skin tissues, skin biopsies were homogenized vigorously in 50 mM phosphate buffer (pH 7.4), and their homogenate was incubated with DCF-DA (10 μM) for 30 min. The level of DCF fluorescence, reflecting the concentration of ROS, was measured at 488 nm excitation and 538 nm emission by a Fluotrac black plate (Greiner BioOne, Frickenhausen, Germany).

### DNA double strand breaks assay

DNA double-strand breaks (DSBs) represent an important ionizing radiation-induced lesion. The rapid phosphorylation of histone H2AX at serine 139 is a sensitive marker for DNA double-strand breaks induced by ionizing radiation which can be later detected by immunofluorescence. HaCaT cells, cultured on coverslips, were fixed in 3.7% paraformaldehyde, permeabilised with 1% Triton X-100 for 20 min at room temperature and then blocked with 1% BSA (Solarbio, Beijing, China) diluted in PBS. Immunofluorescence was performed as described previously using an anti-γH2AX antibody (1:1000, Epitomics, Burlingame, CA, USA) supplemented with 1% BSA and 0.2% Tween-20 for 12 h at 4°C. Followed by TRITC-conjugated anti-rabbit secondary antibody, the cells on the coverslips were counterstained with 4′-6-diamidino-2-phenylindole (DAPI, Invitrogen, Carlsbad, CA, USA) to mark the nuclei. Immunofluorescent staining was observed and photographed using a fluorescence microscope.

### Animals and treatments

Male SD rats (6 weeks old, weight range: 200–300 g) were purchased from Shanghai SLAC Laboratory Animal Co., Ltd. (Shanghai, China). The animals were housed at a constant temperature (23°C) and relative humidity (60%) with a fixed 12-h light:12-h dark cycle and free access to food and water. After anesthetized and shaved hair on rat buttock skin, rats were immobilized with adhesive tape on a plastic plate to minimize motion during irradiation exposure. Three cm thick piece of lead was used to shield the rats and localize the radiation field (3 cm × 4 cm). A single dose of 45 Gy was administered to the treatment area of each rat at a dose rate of 750 cGy/min using a 6-MeV electron beam accelerator (Clinac 2100EX; Varian Medical Systems Inc, CA, USA). This dose was selected because it can significantly induce skin injury as reported [22]. After irradiation, rats were randomly divided into three groups (n = 8 in each group): 1) rats were administered with a subcutaneous injection of 200-μl volume PBS; 2) with a subcutaneous injection of 5,000 U SOD_1_ in a 200-μl volume; 3) with a subcutaneous injection of 5,000 U TAT-SOD_1_ in a 200-μl volume. All the protocols and procedures were approved by the Animal Experimentation Ethics Committee of the Soochow University.

### Malondialdehyde (MDA) concentration measurement

Tissue MDA levels were determined by thiobarbituric acid (TBA) reaction. The optical density (OD) was measured at a wavelength of 532 nm. The assay depended on the measurement of the pink color produced by the interaction of barbituric acid with MDA generated as a result of lipid peroxidation. The colored reaction with 1,1,3,3- tetraethoxy propane was used as the primary standard. Fresh skin samples were homogenized with 50 mM phosphate buffer (pH 7.4). Then, homogenates were centrifuged at 12,000 × g for 10 min at 4°C. MDA levels were expressed as a nanomol per milligram of protein (nmol/mg protein).

### Statistical analysis

Statistical analyses were done with SPSS 16.0 Software (SPSS, Seattle, WA, USA). All data are expressed as mean ± SEM. The experiments were performed at least 3 times. Statistical significance was evaluated by the one-way ANOVA. *P* < 0.05 is considered significant.

## Results

### Preparation of TAT-SOD_1_ and its transduction into HaCaT cells

To investigate the protective role of the TAT-SOD_1_ fusion protein, we constructed a pET-28a-TAT-SOD_1_ expression vector, which contains cDNA sequences encoding the *SOD*_1_ gene and HIV-TAT basic domain (Figure 1A). As shown in Figure 1B, the fusion protein was expressed at a very high level and further induced by IPTG in transformed *E.coli* BL21 cells. The fusion protein was present in the supernatant of the whole cell lysate, indicating that it was soluble in the *E. coli* host cells. Then, the TAT-SOD_1_ and control SOD_1_ fusion protein were purified by affinity chromatography.

We next examined the transduction of TAT-SOD_1_ into HaCaT cells. After seeding, culture medium of HaCaT cells was added with PBS, SOD_1_ or TAT-SOD_1_, respectively. Immunofluorescence analysis revealed that HaCaT cells treated with PBS or natural SOD_1_ showed weak SOD_1_ signal, whereas TAT-SOD_1_ treated HaCaT cells showed increased intracellular localization of SOD_1_ (Figure 1C), indicating transduced exogenous TAT-SOD_1_. These results indicate that TAT-SOD_1_ fusion protein can be transduced into HaCaT cells.

### Increased antioxidant activity and viability of HaCaT cells by TAT-SOD_1_

**Figure 2:**
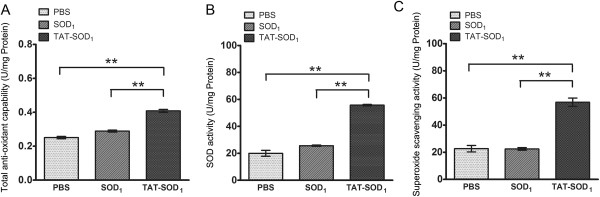
**Bioactivity of transduced TAT-SOD**_**1**_**into cultured HaCaT cells. (A)** Total antioxidant capability, **(B)** SOD activity, **(C)** Superoxide scavenging activity of each group was detected respectively. Each bar in A, B and C represents the mean ± SEM obtained from three experiments. Double asterisks denote a statistically significant difference from control group at *P* < 0 .01.

We next investigated whether TAT-SOD_1_ enhanced antioxidant activity of HaCaT cells. After the addition of TAT-SOD_1_ for 5 h, an average of 62.63% increase of total antioxidant capability was obtained relative to PBS-added cells (*P* < 0.01, Figure 2A). The SOD enzyme activity of cell lysates was significantly elevated in TAT-SOD_1_ treated group, compared with PBS treated group (2.79-fold increase; *P* < 0.01) or SOD_1_ treated group (2.18-fold increase; *P <* 0.01). TAT-SOD_1_ treatment also significantly increased the activity of superoxide scavenging ability compared with PBS treated group (2.51-fold increase; *P <* 0.01) or SOD_1_ treated group (2.54-fold increase; *P* < 0.01). These results demonstrated that TAT-SOD_1_ significantly increased the antioxidant capacity of HaCaT cells.

**Figure 3:**
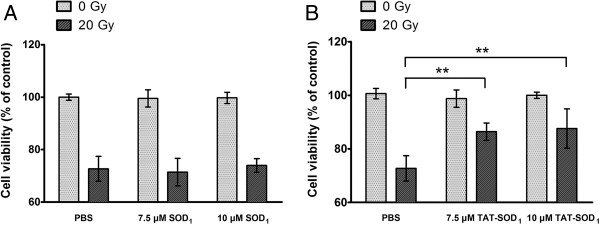
**Effects of TAT-SOD**_**1**_**on cell viability after X-ray irradiation.** HaCaT cells were pretreated with PBS, SOD_1_ or TAT-SOD_1_ for 5 h, respectively. Then, cells were incubated for 48 h. Cell viabilities were determined by with a colorimetric assay using CCK-8. **(A)** Compared with PBS treatment cells, SOD_1_ treatment cells showed no significant difference on cell viability. **(B)** TAT-SOD_1_ treatment cells significantly increased cell viability after X-ray irradiation. *P < 0.05 and **P < 0.01, compared with ionizing radiation alone treated cells. These results are representative of at least three separate experiments.

To explore the effect of TAT-SOD_1_ on cell viability, *in vitro* HaCaT cells were treated with various doses of SOD_1_ or TAT-SOD_1_. SOD_1_ or TAT-SOD_1_ showed no cytotoxicity for non-irradiated HaCaT cells (Figure 3A and 3B). However, pretreatment with TAT-SOD_1_ results in a significant increase of cell viability of HaCaT cells after 20 Gy X-ray irradiation (Figure 3B). While in SOD_1_ pretreated HaCaT cells, the increase of cell viability is less pronounced and is not significant compared with mock treated cells after irradiation (Figure 3A). As expected, cells treated with TAT-SOD_1_ led to a significant increase of cell viability after ionizing radiation relative to cells treated with PBS or SOD_1_.

### Reduced apoptosis of HaCaT cells by TAT-SOD_1_ after X-ray irradiation

**Figure 4:**
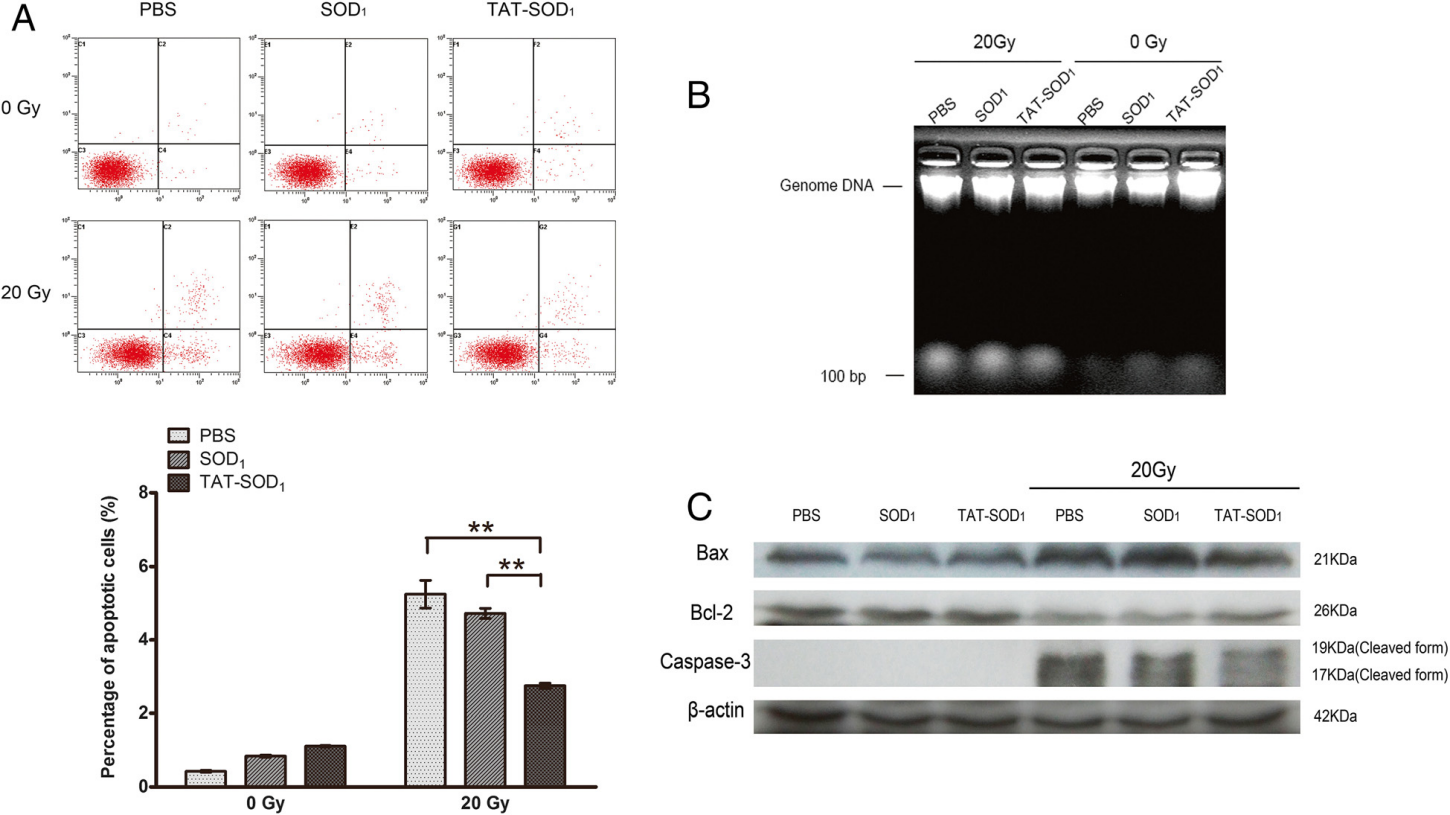
**TAT-SOD**_**1**_**reduces apoptosis of HaCaT cells after X-ray irradiation. (A)** HaCaT cells were treated with PBS, SOD_1_ or TAT-SOD_1_ 5 h prior to irradiation with 20 Gy. Cells were collected 48 h after irradiation, stained with Annexin V-FITC and propidium iodide-TRITC and analyzed by flow cytometry. **(B)** Agarose gel electrophoresis of DNA extracted from HaCaT cells. **(C)** Western blot analysis of apoptotic marker proteins in HaCaT cells exposed to ionizing radiation. Cell extracts were subjected to 12% SDS-PAGE and immunoblotted with antibodies against cleaved Caspase-3, Bcl-2, or Bax. β-Actin was used as an internal control.

Using annexin V and PI staining of live cells, the levels of cell apoptosis after exposure to sham or 20 Gy X-ray irradiation were detected by flow cytometry. The percentage of apoptotic cells in sham irradiated cells was not significantly altered either in the SOD_1_- or TAT-SOD_1_-treated cells compared to the PBS treated cells (Figure 4A). 20 Gy irradiation caused a dramatic increased percentage of apoptotic cells, which was significantly reduced by addition of TAT-SOD_1_. Moreover, DNA fragmentation, a late event in apoptosis, was also induced excessively by irradiation (Figure 4B). However, the ratio of DNA fragmentation was significantly decreased by TAT-SOD_1_, but not by SOD_1_ (Figure 4B). The expression of apoptosis related molecules Bcl-2, Bax and Caspase-3 was further evaluated by Western blot analysis 48 h after irradiation. As shown in Figure 4C, ionizing radiation increased pro-apoptosis proteins Bax, cleaved Caspase-3 and decreased anti-apoptosis protein Bcl-2 expressions. In contrast to PBS or SOD1 treated cells, TAT-SOD_1_ reduced the expression of Bax and cleaved Caspase-3 and increased the expression of Bcl-2 after irradiation. Taken together, these results demonstrated that TAT-SOD_1_ attenuated ionizing radiation-induced apoptosis in HaCaT cells.

### TAT-SOD_1_ maintained mitochondrial integrity of HaCaT cells

**Figure 5:**
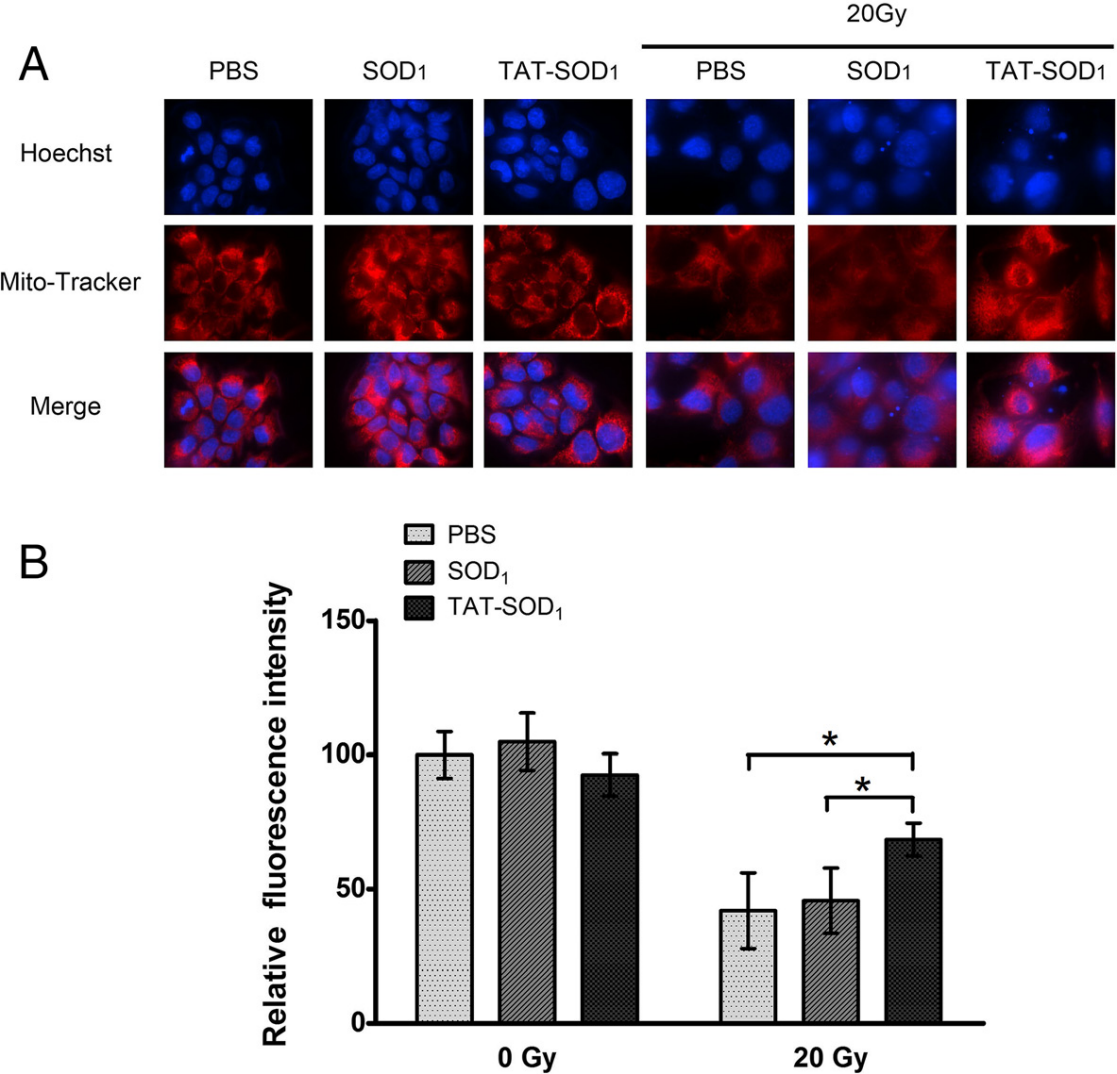
**Effect of TAT-SOD**_**1**_**on mitochondrial integrity of HaCaT cells after ionizing radiation. (A)** Representative confocal images of cellular Mito-Tracker fluorescence 72 h after irradiation with PBS, SOD_1_ or TAT-SOD_1_ treated, respectively. **(B)** Relative fluorescence intensity of mitochondria in HaCaT cells by Image J software (MD, USA).

Free radicals generated from ionizing radiation may damage mitochondrial membrane permeability and change membrane potential, which consequently disrupt mitochondrial functions and induce cells apoptosis [23-25]. To explore the protective role of TAT-SOD_1_ in mitochondrial function, Mito-tracker Red staining was used. As shown in Figure 5A, weak red fluorescence was observed following pretreatment with PBS or SOD_1_ prior to ionizing radiation, indicating destruction of mitochondrial membrane integrity in HaCaT cells. In contrast, TAT-SOD_1_ treatment maintained the strong red fluorescence after 20 Gy X-ray irradiation, suggesting its protection of mitochondria integrity (Figure 5A and 5B). Furthermore, we observed the mitochondrial morphology changed seriously to irregular filamentous in cells pretreated with PBS or SOD_1_ prior to irradiation, while cells pretreated with TAT-SOD_1_ maintained the granular morphology of most mitochondria.

### TAT-SOD_1_ reduced the generation of irradiation-induced intracellular ROS

**Figure 6:**
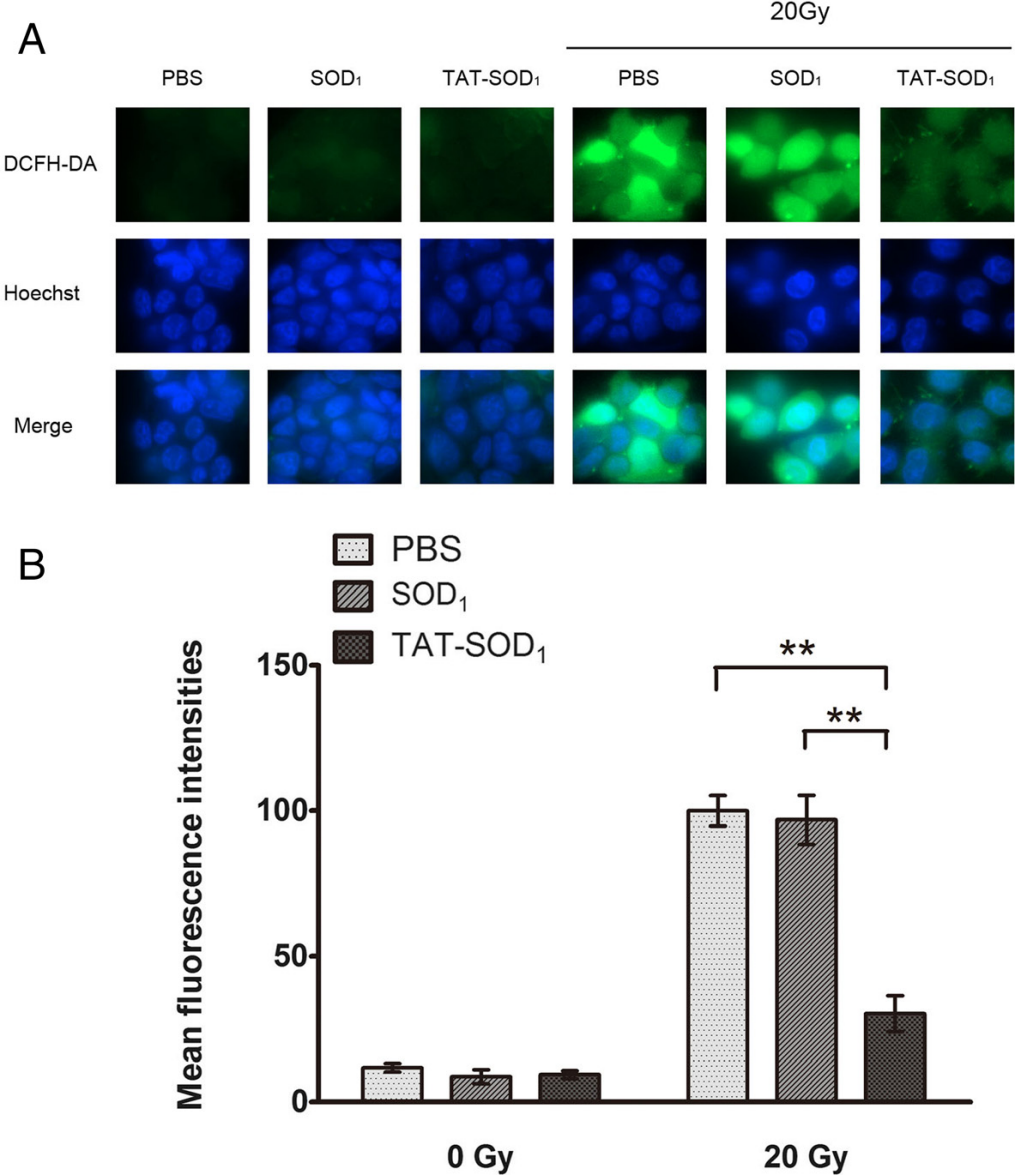
**TAT-SOD**_**1**_**decreased ROS production induced by radiation.** HaCaT cells were pretreated with PBS, SOD_1_ or TAT-SOD_1_ prior to irradiation. **(A)** The representative fluorescence images of ROS levels in HaCaT cells 24 h after exposure to 20 Gy ionizing radiation. **(B)** Quantitative analysis of ROS levels using Image J (MD, USA).

Mitochondrial dysfunction in irradiated cells may significantly contribute to perturbation in oxidation–reduction reactions that determine the cellular redox environment [23,25]. SOD_1_ has been shown to confer cytoprotection against ROS-induced oxidative stress [26]. We therefore investigated whether TAT-coupled SOD_1_ could scavenge radiation-induced intracellular ROS using DCF-DA assay. As shown in Figure 6A and 6B, TAT-SOD_1_ did not affect the background level of cellular ROS. However, compared with PBS or SOD_1_ treated cells, TAT-SOD_1_ reduced ~70% ROS in irradiated HaCaT cells.

### TAT-SOD_1_ decreased the number of γH2AX foci

**Figure 7:**
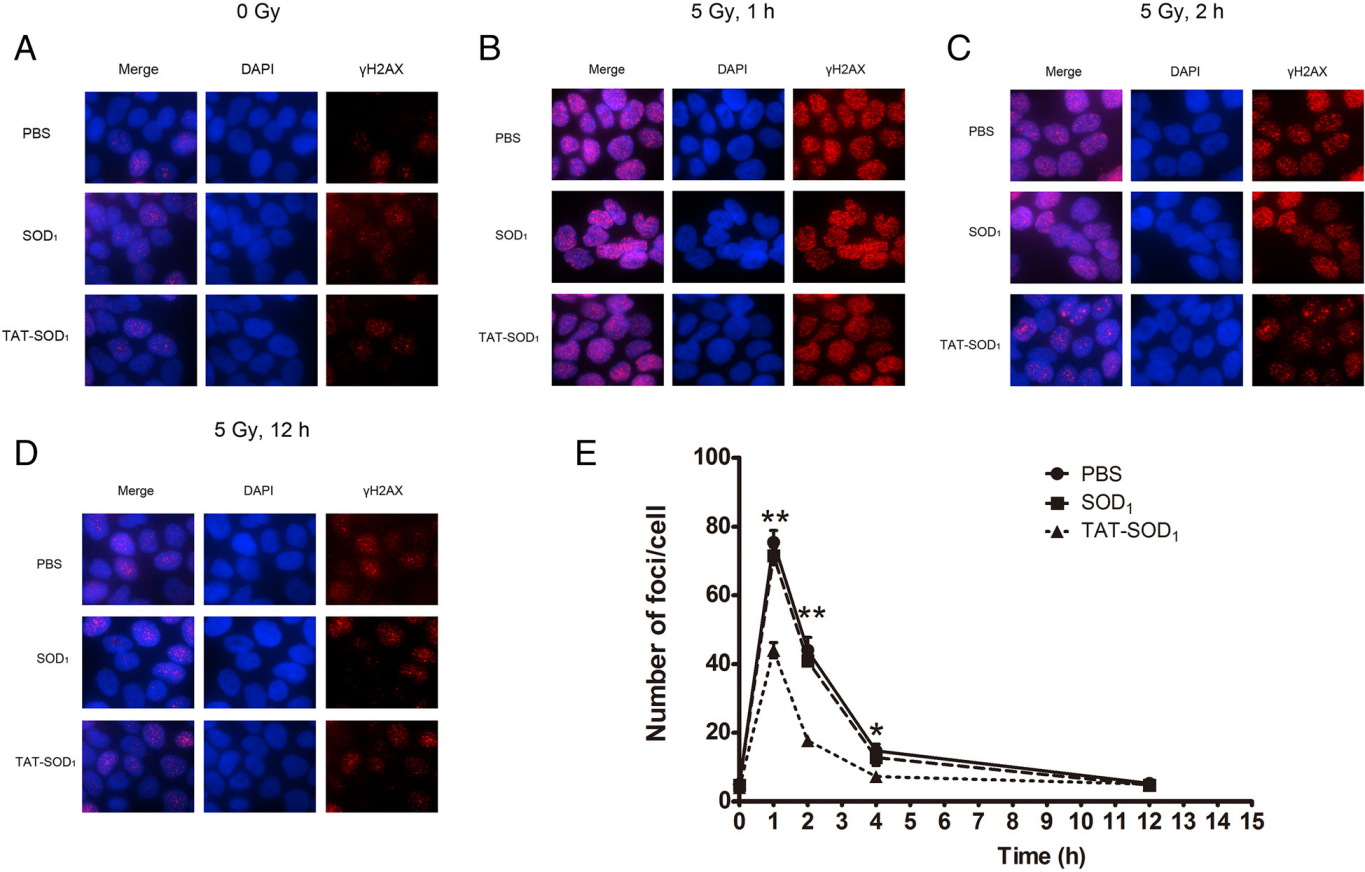
**TAT-SOD**_**1**_**decreased γH2AX foci caused by X-ray irradiation and accelerated DNA double strand breaks repair. (A)** The basic DSB levels of the non-irradiated cells after treatment with PBS, SOD_1_ or TAT-SOD_1_, respectively. **(B)**, **(C)** and **(D)** Representative images of γH2AX foci of PBS, SOD_1_ or TAT-SOD_1_ treated groups after 5 Gy X-ray irradiation at different time points. **(E)** Kinetics of γH2AX foci loss in different groups of cells after 5Gy X-ray irradiation. HaCaT cells were stained for γH2AX at indicated times and then the mean number of γH2AX foci per cell (foci/cell) was counted.

We further explored whether TAT-SOD_1_ would influence the dynamic repair process of DNA double-strand breaks (DSBs) induced by radiation. DNA repair of DSBs was measured by detecting nuclear γH2AX foci at several time points after 5 Gy X-ray irradiation. One hour following 5 Gy irradiation, a rapid induction of γH2AX foci was observed up to more than 70 foci per cell (Figure 7A, 7B and 7E) and then the average number of foci gradually began to decrease until 12 h after radiation exposure (Figure 7D and 7E). The number of foci formed in TAT-SOD_1_-treated cells was significantly reduced to an average of 54.5% (44 foci per cell) of PBS-treated cells at 1 h after irradiation. At 2 and 4 h after irradiation, the number of γH2AX foci in TAT-SOD_1_-treated groups dropped to 40.34% (*P* < 0.01) and 49.15% (*P* < 0.05) of PBS-treated cells, respectively. Comparatively, the SOD_1_-treated cells showed no significant change in the number of γH2AX foci. These results suggested that TAT-SOD_1_ can reduce DSBs induced by ionizing radiation.

### TAT-SOD_1_ treatment reduced ROS generation and MDA content in skin tissue

**Figure 8:**
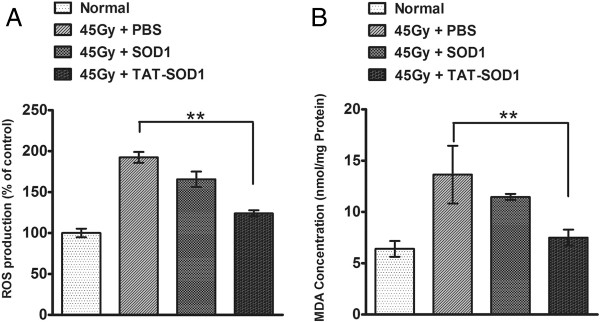
**TAT-SOD**_**1**_**reduced skin ROS and MDA concentration*in vivo*.** Rats were irradiated to the buttock skin with a single dose of 45 Gy electron beams followed by an injection of PBS, SOD_1_ or TAT-SOD_1_. **(A)** The ROS generation in rat skin tissues of indicated groups 24 h after 45 Gy electron beam irradiation. The basic ROS level of the control group was arbitrarily set as 100%. **(B)** Relative MDA concentration in rat skin tissues of indicated groups 3 days after 45 Gy electron beam irradiation. *P < 0.05; **P < 0.01, compared with PBS-injected control groups.

We next investigated whether TAT-SOD_1_ could eliminate ROS in skin tissues. As shown in Figure 8A, 45 Gy of electron beam irradiation caused a significant increase in ROS level and MDA content in rat skin tissues. No significant differences of ROS and MDA levels were observed between the SOD_1_-treated group and the control skins of rats. Comparatively, a significant decrease in ROS level and MDA concentration was observed in the group treated with TAT-SOD_1_ (*P* < 0.01). These results indicated that administration of TAT-SOD_1_ attenuated radiation-induced ROS generation and lipid peroxidation *in vivo*.

## Discussion

SODs have been used as potential therapeutic agents for ROS-mediated diseases, however, the disadvantage of the ineffective delivery into cells and tissues limited their application [27,28]. All the major efforts in utilizing SOD for radioprotection focus on the modification of the molecule so that it can be delivered into the cells, the methods of overexpression SOD_1_ intracellular including liposomal encapsulation, plasmid transfection or adenovirus-mediated infection [26,29-31]. An alternative approach that appears to be safer is to produce recombinant proteins and then to inject them into individual target cells by micro-injectors. However, there are several drawbacks of these methods: for example, the efficiency of the target gene uptake by viral vector-mediated methods is unstable and it would take more than 24 h for expression of target genes [32,33]. The introduction of exogenous genes may integrate into host genomes, which may interfere with the intact genes of the host. Under normal physiological conditions, only a few small molecules can freely pass through eukaryotic cell membranes, while proteins and other large biological macromolecules are seldomly transported into cells, making it difficult for the treatment of diseases [34]. Although the mechanism of protein transduction is not completely clear, it is generally accepted that the PTDs across cell membrane efficiently. The protein transduction mediated by HIV TAT PTD speed the application of target proteins, which does not alter the host genome. Many previous results showed that it takes only several hours for HIV TAT PTD carried molecules to enter the cells and HIV TAT shows minimal cell toxicity and influence on bioavailability of macromolecules. In addition, the delivery amount and the time of incubation could be controlled precisely [35,36].

Skin is the first organ to be targeted by external radiations and among the most sensitive tissues. High doses of radiation can induce various deleterious effects in skin such as erythema and desquamation in a short time, and several years later stochastic effects such as skin carcinoma may occur [2]. Effective suppression of radiation-induced skin damage is beneficial for radiotherapy of cancers. Therefore, methods to reduce skin damage induced by ionizing radiation are warranted. In this study, we purified a cell membrane permeable SOD, TAT-SOD_1_ from bacterial cells. The recombinant TAT-SOD_1_ protein overcame the shortcomings of natural SODs, which could be rapidly and efficiently transported into cultured HaCaT cells and delivered in the cytoplasm and cell nucleus. Further functional experiments confirmed that the fusion protein maintained biological activity and can significantly inhibit cell death induced by ionizing radiation. Pretreatment of TAT-SOD_1_ prevented the HaCaT cells from ionizing radiation-induced injury by decreasing ROS, apoptosis, DNA DSBs and mitochondria damage. Moreover, TAT-SOD_1_ attenuated ROS and MDA concentration in irradiated rat skins.

Ionizing radiation generates cellular ROS, which have high potential to damage cellular macromolecules including proteins, lipids, and nucleic acids [37-39]. Recent evidence suggests that ROS plays an important role in cell death and signal transduction after ionizing radiation. TAT-SOD_1_ can obviously reduce intracellular ROS levels, which is mainly due to its ability to cross the cell membrane. Mitochondrial are vulnerable to oxidants because they are the major source of free radicals in the cells and limited to their ability to cope with oxidative stresses [40]. Mitochondrial is closely related to cell apoptosis and the destruction of the mitochondrial transmembrane potential is considered to be one of the earliest events in the process of apoptosis cascade and occurs earlier than the apoptosis characterized in the nucleus (chromatin condensation and DNA fragmentation) [41]. ROS generated from mitochondria contribute to genomic instability after exposure of the cells to ionizing radiation [40,42]. It is reported that a variety of antioxidants such as mangiferin and red ginseng can reduce mitochondrial apoptosis pathway dependent cell death [43,44]. In this study, we showed that TAT-SOD_1_ pretreatment, but not natural SOD_1_, was able to mitigate mitochondrial dysfunction. TAT-SOD_1_ also reduced the mitochondrial dysfunction dependent cell death in HaCaT cells. The Bcl-2 family proteins play a pivotal role in the regulation of mitochondrial apoptotic pathways [45]. Following initiation of mitochondria-dependent apoptosis by Bax, the activated caspase-3 and effector molecules result in cellular apoptosis. Overexpression of Bcl-2 can inhibit apoptosis [46]. In our study, the protein expression levels of anti-apoptotic member Bcl-2 were increased and pro-apoptotic member Bax were reduced when treated with TAT-SOD_1_ prior to X-ray irradiation, which is accompanied by a reduced level of active Caspase-3. The DNA fragmentation assay also showed the decreased ionizing radiation induced cell apoptosis in TAT-SOD1 treated cells. Moreover, TAT-SOD_1_ can also decrease the γH2AX foci, which is a biomarker of radiation-induced DSBs. DNA DSBs caused by irradiation tend to impair the integrity of the genome and cause cell death [47]. The DNA damage effect is thought to be mediated partially by the direct effects of radiation-induced ionization and partially by ROS generated by ionization of water [48]. Radiation-induced DNA impairment was alleviated in TAT-SOD_1_ treated cells, which is possibly attributed to the significantly decreased intracellular ROS levels. Taken together, these results indicate that transduced TAT-SOD_1_ protein plays a defensive role against cell death induced by oxidative stress in skin cells.

We also detected the radiation protective effect of TAT-SOD_1_*in vivo*. Radiation exposure to skin tissues generates ROS and oxidative stress, which will trigger an inflammatory response and cell death in affected areas. The ROS is known to oxidize fatty acids generating highly toxic lipid peroxides that lead to apoptotic cell death. In the present study, we demonstrated that injection of TAT-SOD_1_ reduced radiation-induced skin MDA levels indicating that TAT-SOD_1_ attenuates radiation-induced lipid peroxidation. The *in vivo* skin protective function of TAT-SOD_1_ was possibly due to its’ characteristics of membrane permeability and intracellular scavenging capability of ROS.

## Conclusions

In summary, we demonstrated that Cu/Zn-SOD (SOD_1_), one of the key intracellular antioxidant enzymes, can be incorporated into HaCaT cells by TAT PTD-mediated protein transduction *in vitro*. Compared with natural SOD_1_, the application of TAT-SOD_1_ significantly maintained mitochondrial integrity and reduced ROS, apoptosis and γH2AX foci after X-ray irradiation in HaCaT cells. Furthermore, administration of TAT-SOD_1_ resulted in a significant decrease in ROS and MDA concentration in electron beam irradiated rat skins. These results suggest that the transduction of TAT-SOD_1_ offers a new strategy for protecting skin against ionizing radiation.

## Competing interests

The authors declare that they have no competing interests.

## Authors’ contributions

QG and TF carried out the molecular biology studies and drafted the manuscript. YT and JX performed the animal experiment. XG, JL and HY performed the statistical analysis. SZ, JC and CH participated in study design and coordination and helped to draft the manuscript. All authors read and approved the final manuscript.

## References

[B1] Baskar R Lee KA Yeo R Yeoh KW Cancer and radiation therapy: current advances and future directions Int J Med Sci 2012 9 193 199 10.7150/ijms.3635 22408567 PMC3298009

[B2] Ryan JL Ionizing radiation: the good, the bad, and the ugly J Invest Dermatol 2012 132 985 993 10.1038/jid.2011.411 22217743 PMC3779131

[B3] Riley PA Free radicals in biology: oxidative stress and the effects of ionizing radiation Int J Radiat Biol 1994 65 27 33 10.1080/09553009414550041 7905906

[B4] Liochev SI Fridovich I Mechanism of the peroxidase activity of Cu, Zn superoxide dismutase Free Radic Biol Med 2010 48 1565 1569 10.1016/j.freeradbiomed.2010.02.036 20211248

[B5] Halliwell B Gutteridge JM Role of free radicals and catalytic metal ions in human disease: an overview Methods Enzymol 1990 186 1 85 2172697 10.1016/0076-6879(90)86093-b

[B6] Jin LH Bahn JH Eum WS Kwon HY Jang SH Han KH Kang TC Won MH Kang JH Cho SW Transduction of human catalase mediated by an HIV-1 TAT protein basic domain and arginine-rich peptides into mammalian cells Free Radic Biol Med 2001 31 1509 1519 10.1016/S0891-5849(01)00734-1 11728823

[B7] Eum WS Choung IS Li MZ Kang JH Kim DW Park J Kwon HY Choi SY HIV-1 Tat-mediated protein transduction of Cu, Zn-superoxide dismutase into pancreatic beta cells in vitro and in vivo Free Radic Biol Med 2004 37 339 349 10.1016/j.freeradbiomed.2004.04.036 15223067

[B8] Kim DW Eum WS Jang SH Kim SY Choi HS Choi SH An JJ Lee SH Lee KS Han K Transduced Tat-SOD fusion protein protects against ischemic brain injury Mol Cells 2005 19 88 96 15750345

[B9] Kimura H Minakami H Otsuki K Shoji A Cu-Zn superoxide dismutase inhibits lactate dehydrogenase release and protects against cell death in murine fibroblasts pretreated with ultraviolet radiation Cell Biol Int 2000 24 459 465 10.1006/cbir.2000.0513 10875893

[B10] Wu Y Ren C Gao Y Hou B Chen T Zhang C A novel method for promoting heterologous protein expression in Escherichia coli by fusion with the HIV-1 TAT core domain Amino Acids 2010 39 811 820 10.1007/s00726-010-0534-2 20213440

[B11] Frankel AD Pabo CO Cellular uptake of the tat protein from human immunodeficiency virus Cell 1988 55 1189 1193 10.1016/0092-8674(88)90263-2 2849510

[B12] Green M Loewenstein PM Autonomous functional domains of chemically synthesized human immunodeficiency virus tat trans-activator protein Cell 1988 55 1179 1188 10.1016/0092-8674(88)90262-0 2849509

[B13] Fawell S Seery J Daikh Y Moore C Chen LL Pepinsky B Barsoum J Tat-mediated delivery of heterologous proteins into cells Proc Natl Acad Sci USA 1994 91 664 668 10.1073/pnas.91.2.664 8290579 PMC43009

[B14] Schwarze SR Ho A Vocero-Akbani A Dowdy SF In vivo protein transduction: delivery of a biologically active protein into the mouse Science 1999 285 1569 1572 10.1126/science.285.5433.1569 10477521

[B15] Watson K Edwards RJ HIV-1-trans-activating (Tat) protein: both a target and a tool in therapeutic approaches Biochem Pharmacol 1999 58 1521 1528 10.1016/S0006-2952(99)00209-9 10535742

[B16] Wadia JS Dowdy SF Protein transduction technology Curr Opin Biotechnol 2002 13 52 56 10.1016/S0958-1669(02)00284-7 11849958

[B17] Zhang X Wang F Intracellular transduction and potential of Tat PTD and its analogs: from basic drug delivery mechanism to application Expert Opin Drug Deliv 2012 9 457 472 10.1517/17425247.2012.663351 22432469

[B18] Spitere K Toulouse A O’Sullivan DB Sullivan AM TAT-PAX6 protein transduction in neural progenitor cells: a novel approach for generation of dopaminergic neurones in vitro Brain Res 2008 1208 25 34 18387597 10.1016/j.brainres.2008.02.065

[B19] Ye N Liu S Lin Y Rao P Protective effects of intraperitoneal injection of TAT-SOD against focal cerebral ischemia/reperfusion injury in rats Life Sci 2011 89 868 874 10.1016/j.lfs.2011.09.015 21983418

[B20] Hernandez Losa J Parada Cobo C Guinea Viniegra J Sanchez-Arevalo Lobo VJ Ramon y Cajal S Sanchez-Prieto R Role of the p38 MAPK pathway in cisplatin-based therapy Oncogene 2003 22 3998 4006 10.1038/sj.onc.1206608 12821934

[B21] Boukamp P Popp S Bleuel K Tomakidi E Burkle A Fusenig NE Tumorigenic conversion of immortal human skin keratinocytes (HaCaT) by elevated temperature Oncogene 1999 18 5638 5645 10.1038/sj.onc.1202934 10523843

[B22] Zhang S Song C Zhou J Xie L Meng X Liu P Cao J Zhang X Ding WQ Wu J Amelioration of radiation-induced skin injury by adenovirus-mediated heme oxygenase-1 (HO-1) overexpression in rats Radiat Oncol 2012 7 4 10.1186/1748-717X-7-4 22247972 PMC3282628

[B23] Kim EH Sohn S Kwon HJ Kim SU Kim MJ Lee SJ Choi KS Sodium selenite induces superoxide-mediated mitochondrial damage and subsequent autophagic cell death in malignant glioma cells Cancer Res 2007 67 6314 6324 10.1158/0008-5472.CAN-06-4217 17616690

[B24] Lemasters JJ Nieminen AL Qian T Trost LC Elmore SP Nishimura Y Crowe RA Cascio WE Bradham CA Brenner DA The mitochondrial permeability transition in cell death: a common mechanism in necrosis, apoptosis and autophagy Biochim Biophys Acta 1998 1366 177 196 10.1016/S0005-2728(98)00112-1 9714796

[B25] Yang J Wu LJ Tashino S Onodera S Ikejima T Reactive oxygen species and nitric oxide regulate mitochondria-dependent apoptosis and autophagy in evodiamine-treated human cervix carcinoma HeLa cells Free Radic Res 2008 42 492 504 10.1080/10715760802112791 18484413

[B26] Freeman BA Young SL Crapo JD Liposome-mediated augmentation of superoxide dismutase in endothelial cells prevents oxygen injury J Biol Chem 1983 258 12534 12542 6688807

[B27] Lefaix JL Delanian S Leplat JJ Tricaud Y Martin M Nimrod A Baillet F Daburon F Successful treatment of radiation-induced fibrosis using Cu/Zn-SOD and Mn-SOD: an experimental study Int J Radiat Oncol Biol Phys 1996 35 305 312 10.1016/0360-3016(96)00061-2 8635938

[B28] Epperly M Bray J Kraeger S Zwacka R Engelhardt J Travis E Greenberger J Prevention of late effects of irradiation lung damage by manganese superoxide dismutase gene therapy Gene Ther 1998 5 196 208 10.1038/sj.gt.3300580 9578839

[B29] Delanian S Baillet F Huart J Lefaix JL Maulard C Housset M Successful treatment of radiation-induced fibrosis using liposomal Cu/Zn superoxide dismutase: clinical trial Radiother Oncol 1994 32 12 20 10.1016/0167-8140(94)90444-8 7938674

[B30] Tsubokawa T Jadhav V Solaroglu I Shiokawa Y Konishi Y Zhang JH Lecithinized superoxide dismutase improves outcomes and attenuates focal cerebral ischemic injury via antiapoptotic mechanisms in rats Stroke 2007 38 1057 1062 10.1161/01.STR.0000257978.70312.1d 17272760

[B31] Reddy MK Labhasetwar V Nanoparticle-mediated delivery of superoxide dismutase to the brain: an effective strategy to reduce ischemia-reperfusion injury FASEB J 2009 23 1384 1395 10.1096/fj.08-116947 19124559

[B32] Perrone S Usai M Lazzari P Tucker SJ Wallace HM Zanda M Efficient cell transfection with melamine-based gemini surfactants Bioconjug Chem 2013 24 176 187 10.1021/bc3004292 23297813

[B33] Biswas J Mishra SK Kondaiah P Bhattacharya S Syntheses, transfection efficacy and cell toxicity properties of novel cholesterol-based gemini lipids having hydroxyethyl head group Org Biomol Chem 2011 9 4600 4613 10.1039/c0ob00940g 21537498

[B34] Hou CL Huang Q Wei Y Zhang W Mi JH Ying DJ Zhou ZH Protein transduction domain-hA20 fusion protein protects endothelial cells against high glucose-induced injury Genet Mol Res 2012 11 1899 1908 10.4238/2012.July.19.9 22869545

[B35] Suhorutsenko J Oskolkov N Arukuusk P Kurrikoff K Eriste E Copolovici DM Langel U Cell-penetrating peptides, PepFects, show no evidence of toxicity and immunogenicity in vitro and in vivo Bioconjug Chem 2011 22 2255 2262 10.1021/bc200293d 21978260

[B36] Stewart KM Horton KL Kelley SO Cell-penetrating peptides as delivery vehicles for biology and medicine Org Biomol Chem 2008 6 2242 2255 10.1039/b719950c 18563254

[B37] Gutowski M Kowalczyk S A study of free radical chemistry: their role and pathophysiological significance Acta Biochim Pol 2013 60 1 16 23513192

[B38] Gollapalle E Wang R Adetolu R Tsao D Francisco D Sigounas G Georgakilas AG Detection of oxidative clustered DNA lesions in X-irradiated mouse skin tissues and human MCF-7 breast cancer cells Radiat Res 2007 167 207 216 10.1667/RR0659.1 17390728

[B39] Rugo RE Schiestl RH Increases in oxidative stress in the progeny of X-irradiated cells Radiat Res 2004 162 416 425 10.1667/RR3238 15447041

[B40] Yoshida T Goto S Kawakatsu M Urata Y Li TS Mitochondrial dysfunction, a probable cause of persistent oxidative stress after exposure to ionizing radiation Free Radic Res 2012 46 147 153 10.3109/10715762.2011.645207 22126415

[B41] Keeble JA Gilmore AP Apoptosis commitment–translating survival signals into decisions on mitochondria Cell Res 2007 17 976 984 10.1038/cr.2007.101 18071367

[B42] Kamat JP Devasagayam TP Oxidative damage to mitochondria in normal and cancer tissues, and its modulation Toxicology 2000 155 73 82 10.1016/S0300-483X(00)00279-1 11154799

[B43] Pal PB Sinha K Sil PC Mangiferin, a natural xanthone, protects murine liver in pb(II) induced hepatic damage and cell death via MAP kinase, NF-kappaB and Mitochondria Dependent Pathways PLoS One 2013 8 e56894 10.1371/journal.pone.0056894 23451106 PMC3581562

[B44] Dong GZ Jang EJ Kang SH Cho IJ Park SD Kim SC Kim YW Red ginseng abrogates oxidative stress via mitochondria protection mediated by LKB1-AMPK pathway BMC Complement Altern Med 2013 13 64 10.1186/1472-6882-13-64 23506615 PMC3635924

[B45] Huang Z Bcl-2 family proteins as targets for anticancer drug design Oncogene 2000 19 6627 6631 10.1038/sj.onc.1204087 11426648

[B46] Byrne GI Ojcius DM Chlamydia and apoptosis: life and death decisions of an intracellular pathogen Nat Rev Microbiol 2004 2 802 808 10.1038/nrmicro1007 15378044

[B47] Ngwa W Korideck H Kassis AI Kumar R Sridhar S Makrigiorgos GM Cormack RA In vitro radiosensitization by gold nanoparticles during continuous low-dose-rate gamma irradiation with I-125 brachytherapy seeds Nanomedicine 2013 9 25 27 10.1016/j.nano.2012.09.001 23041410 PMC3723694

[B48] Niu Y Wang H Wiktor-Brown D Rugo R Shen H Huq MS Engelward B Epperly M Greenberger JS Irradiated esophageal cells are protected from radiation-induced recombination by MnSOD gene therapy Radiat Res 2010 173 453 461 10.1667/RR1763.1 20334517 PMC2872095

